# Plasma levels of alpha1-antichymotrypsin and secretory leukocyte proteinase inhibitor in healthy and chronic obstructive pulmonary disease (COPD) subjects with and without severe α1-antitrypsin deficiency

**DOI:** 10.1186/1471-2466-7-1

**Published:** 2007-01-29

**Authors:** Camilla Hollander, Ulla Westin, Anders Wallmark, Eeva Piitulainen, Tomas Sveger, Sabina M Janciauskiene

**Affiliations:** 1 Departments of Otolaryngology and Head and Neck Surgery, Institution of Clinical Sciences, Lund University, University Hospital Malmö, SE-205 02 Malmö, Sweden; 2 Department of Medicine, Institution of Clinical Sciences, Lund University, University Hospital Malmö, SE-205 02 Malmö, Sweden; 3 Department of Pediatrics, Institution of Clinical Sciences, Lund University University Hospital Malmö, SE-205 02 Malmö, Sweden

## Abstract

**Background:**

Individuals with severe Z α1-antitrypsin (AAT) deficiency have a considerably increased risk of developing chronic obstructive lung disease (COPD). It has been hypothesized that compensatory increases in levels of other protease inhibitors mitigate the effects of this AAT deficiency. We analysed plasma levels of AAT, α1-antichymotrypsin (ACT) and secretory leukocyte protease inhibitor (SLPI) in healthy (asymptomatic) and COPD subjects with and without AAT deficiency.

**Methods:**

Studied groups included: 71 asymptomatic AAT-deficient subjects (ZZ, n = 48 and SZ, n = 23, age 31 ± 0.5) identified during Swedish neonatal screening for AAT deficiency between 1972 and 1974; age-matched controls (MM, n = 57, age 30.7 ± 0.6); older asymptomatic ZZ (n = 10); healthy MM (n = 20, age 53 ± 9.6); and COPD patients (ZZ, n = 10, age 47.4 ± 11 and MM, n = 10, age 59.4 ± 6.7). Plasma levels of SLPI, AAT and ACT were analysed using ELISA and immunoelectrophoresis.

**Results:**

No significant difference was found in plasma ACT and SLPI levels between the healthy MM and the ZZ or SZ subjects in the studied groups. Independent of the genetic variant, subjects with COPD (n = 19) had elevated plasma levels of SLPI and ACT relative to controls (n = 153) (49.5 ± 7.2 vs 40.7 ± 9.1 ng/ml, p < 0.001 and 0.52 ± 0.19 vs 0.40 ± 0.1 mg/ml, p < 0.05, respectively).

**Conclusion:**

Our findings show that plasma levels of ACT and SLPI are not elevated in subjects with genetic AAT deficiency compared MM controls and do not appear to compensate for the deficiency of plasma AAT.

## Background

Serine proteases play key roles in coagulation, fibrinolysis, and in kinin and complement activation. The activities of these enzymes are controlled at least in part by specific serine protease inhibitors, most of which are serpins. The relationship between serine protease and serpin inhibitor levels has been examined extensively, since an imbalance between them is linked to local tissue injury and to many pathologies, including cancer, autoimmune diseases, chronic obstructive lung disease (COPD), inflammation and infectious diseases [1-5].

The serine protease inhibitor found in highest concentration in plasma is α1-antitrypsin (AAT). Severe AAT deficiency in the homozygous Z variant, which differs from the wild type M variant in the substitution of Glu-342 by Lys, was first recognized as a hereditary condition predisposing to COPD on the basis of low plasma levels (10% of normal) of AAT [6]. This single amino acid mutation perturbs the folding and tertiary structure of AAT, leading to spontaneous polymerization and cellular retention. Thus, it is a failure in the secretion rather than biosynthesis of AAT that leads to the deficiency [6,7]. AAT is mainly synthesized by liver cells and the Z variant of AAT is retained as inclusion bodies in the endoplasmic reticulum of hepatocytes[8]. These Z-AAT polymers are hypothesized to be cytotoxic and cause liver damage with variable clinical presentation ranging from neonatal cholestasis to liver cirrhosis and hepatocellular carcinoma in adults [9-11].

Individuals with severe AAT deficiency have at least a 20-fold increased risk of developing lung disease, especially if they smoke [12]. AAT is the most important inhibitor of neutrophil elastase and proteinase 3, and it was suggested that proteases from activated neutrophils, inadequately regulated by AAT, cause destruction of lung tissue [13]. Other serine protease inhibitors, such as α1-antichymotrypsin (ACT), α2-macroglobulin (α2 MG), antithrombin and antiplasmin, also play important roles in controlling serine protease activity. While these latter inhibitors are produced mainly by the liver and reach tissues by passive diffusion [14], other serine protease inhibitors, such as Secretory Leukocyte Proteinase Inhibitor (SLPI) and elafin/SKALP, are produced locally by airway epithelial cells [15,16]. It was proposed that SLPI, as a major anti-elastase inhibitor of the bronchi, is important for protecting the respiratory epithelium [17,18]. In contrast to AAT, SLPI blocks elastin-bound elastase in the alveolar walls, which might also protect against the development of COPD [19].

It has been hypothesized that compensatory increases in other protease inhibitors and/or decreased leukocyte activity may reduce the severity of AAT deficiency by favourably affecting the overall protease/protease-inhibitor balance in AAT-deficient individuals [20-22]. To further test this hypothesis that compensating increases of other protease inhibitor concentrations occur in cases with inherited AAT deficiency, we determined plasma AAT, ACT and SLPI levels in different age groups of healthy and COPD adult subjects with and without AAT deficiency.

## Methods

### Study subjects

The first study group included 71 asymptomatic, 31 year old, AAT-deficient subjects: ZZ, n = 48, 24 females and 24 males, age 31.1 ± 0.52 years and SZ, n = 23, 12 females and 11 males, age 31 ± 0.58 years, identified in the Swedish neonatal screening study during 1972–1974 [20-22]. Clinical examination, spirometry, and routine blood tests were performed by a chest physician at the local hospitals. An age-matched control group MM (n = 57, 33 females and 24 males, age 31.7 ± 0.6 years) was recruited from the general population living in the south of Sweden. No significant differences in lung function (FEV_1 _and FEV_1_/FVC%) was found among the studied subject groups with ZZ, SZ and MM variants of AAT.

The second study group consisted of 20 healthy MM AAT adults (11 males and 9 females, aged 53 ± 9.6 years), 10 asymptomatic ZZ AAT adults (5 males and 5 females, aged 53 ± 9.6 years) and 20 COPD patients, among them 10 patients with ZZ AAT (5 males and 5 females, aged 47.4 ± 11 years) and 10 patients with MM AAT (5 males and 5 females, aged 59.4 ± 6.7 years). COPD was diagnosed according to the NHLBI/WHO Workshop guidelines [23]. The ZZ and MM COPD patients had a forced expiratory volume in one second (FEV1) ≤ 80% of that predicted and a FEV1/Forced vital capacity ratio (FVC) ≤ 70%, while asymptomatic ZZ individuals had normal lung function. All COPD patients included in the study were in a stable, non-exacerbated phase of the disease. The exclusion criteria were liver diseases, vasculitic or other extra-pulmonary diseases. The control subjects showed no evidence of any disease and had no respiratory symptoms; none of them was on medication and all had MM variant and normal plasma concentration of AAT. The ZZ individuals were recruited from the Swedish AAT Deficiency Register. The MM COPD individuals were outpatients at the Department of Respiratory Medicine, University Hospital, Malmo. The healthy volunteers were recruited from the hospital staff and their relatives. All individuals gave a signed, informed consent to take part in this study, which has been approved by the research ethical committee of Lund University, Sweden.

### Blood sample collection

Blood was taken by venipuncture, plasma was directly separated by centrifugation and stored at -20°C or -80°C until assayed. AAT-phenotyping was performed by isoelectric focusing at the Department of Clinical Chemistry, University Hospital.

### Assay of SLPI

Secretory leukocyte proteinase inhibitor (SLPI) was analysed using ELISA kits (R&D Systems Europe Ltd, Abingdon, UK) according to the manufacturer's instructions. Absorbance was measured spectrophotometrically at 450 nm using a microplate reader (Labsystems). The minimum detectable level of SLPI was 0, 0625 ng/ml.

### Quantitative analysis of ACT and AAT

The plasma concentration of ACT and AAT was determined by the rocket immunoelectrophoresis method based on a quantitative estimation of proteins by electrophoresis in 1% agarose gel containing monospecific antibodies against ACT or AAT at a concentration of 5.5 μg per square gel area [23]. The ACT concentration was quantified by counting the height of the rocket-shaped precipitation zone. For the calibration curve a standard plasma Seronorm™ with a known concentration of ACT and AAT was used.

### Statistical analysis

The statistical package SPSS for Windows (release 11.5, SPSS Inc., Chicago) was used for statistical calculations. Differences in the means were analysed for their statistical significance with the one-way ANOVA combined with a multiple-comparisons procedure (Scheffe multiple range test). The equality of means was analysed for statistical significance with an independent two sample t-test and Pearson correlation analysis. Tests showing p < 0.05 were considered to be significant. Data are expressed as mean ± SD.

## Results

### Plasma levels of serine protease inhibitors in 31 year old asymptomatic AAT-deficiency and age and gender matched MM AAT subjects

As expected, plasma levels of AAT in 31 year old subjects were ranked: MM>SZ>ZZ and are significantly different among the groups [F(2/125) = 216, p < 0.001] (Table 1). However, we found no significant difference in plasma ACT and SLPI levels between subjects with deficiency of AAT and subjects with wild type AAT (age and gender matched controls)

**Table 1:** Plasma levels of serine protease inhibitors in 31 year old asymptomatic AAT-deficiency and age and gender matched wild-type AAT subjects.

AAT-variant	N	F/M	AAT (mg/ml)	ACT (mg/ml)	SLPI (ng/ml)
			Mean ± SD	Mean ± SD	Mean ± SD
ZZ	48	24/24	0.27 ± 0.05	0.41 ± 0.11	40.2 ± 9.6
SZ	23	12/11	0.64 ± 0.14	0.39 ± 0.08	40.7 ± 8.7
MM	57	33/24	1.44 ± 0.43	0.38 ± 0.08	41.0 ± 7.6

N-number of subjects

SD, standard deviation

### Plasma levels of serine protease inhibitors in older healthy (asymptomatic) and COPD subjects with and without severe Z AAT deficiency

We found no significant difference in plasma levels of SLPI and ACT between deficiency and normal AAT subjects in the absence of COPD (Table 2A). For the COPD cases (Table 2B), the only difference between normal and AAT deficiency groups is a marginally (within standard deviation) higher plasma ACT level in the cases with MM AAT compared to those with ZZ AAT [t(17) = 2.5, p < 0.05)].

**Table 2:** Plasma levels of serine protease inhibitors in older healthy (asymptomatic) and COPD subjects with and without severe Z AAT deficiency.

**Healthy (asymptomatic) subjects (A)**						
AAT-variant	N	AAT (mg/ml)	N	ACT (mg/ml)	N	SLPI (ng/ml)
		Mean* ± SD		Mean* ± SD		Mean* ± SD

ZZ	10	0.20 ± 0.03	10	0.40 ± 0.20	9	44.0 ± 15
MM	20	1.37 ± 0.20	15	0.37 ± 0.15	13	38.2 ± 9.9

**COPD subjects (B)**						

AAT-variant	N	AAT (mg/ml)	N	ACT (mg/ml)	N	SLPI (ng/ml)
		Mean* ± SD		Mean* ± SD		Mean* ± SD

ZZ	10	0.32 ± 0.17	10	0.42 ± 0.12	10	48.5 ± 8.4
MM	10	1.73 ± 0.22	9	0.62 ± 0.21	9	51.1 ± 6.1

N-subject number

* geometric mean value

SD, standard deviation

### Plasma levels of serine protease inhibitors in healthy (asymptomatic) and COPD subjects independent of genetic variant of AAT and age

It is noteworthy that AAT levels were similar in all asymptomatic ZZ AAT cases independent of age (0.27 ± 0.05 mg/ml, n = 48, mean age 32 years and 0.2 ± 0.03 mg/ml, n = 10, mean age 53 years), while ZZ COPD patients had 25% higher plasma AAT levels compared to asymptomatic ZZ cases, although this was not statistically significant. We also compared levels of ACT and SLPI in healthy/asymptomatic and COPD individuals independent of the genetic variant of AAT and age. As shown in Table 3, levels of both protease inhibitors were found to be elevated in COPD patients relative to healthy subjects (ACT t(19.4) = 2.8, p < 0.05 and SLPI t(167) = 4.2, p < 0.001). Previous studies have also demonstrated increased levels of ACT, SLPI and α2-MG in COPD [24-27].

**Table 3:** Plasma levels of serine protease inhibitors in asymptomatic and COPD subjects independent of genetic variant of AAT

Subjects	N	ACT (mg/ml)	N	SLPI (ng/ml)
		Mean* ± SD		Mean* ± SD
Healthy	153	0.40 ± 0.10	153	40.7 ± 9.1
COPD	19	0.52 ± 0.19	20	49.5 ± 7.2

*) geometric mean

SD, standard deviation

N-number of subjects

## Discussion

To contain the potential injurious effects of serine proteases, the anti-proteases developed in a parallel network consisting of "alarm", locally produced such as SLPI and elafin/ESI/SKALP, and "systemic" inhibitors. The latter, such as α2-MG, ACT and AAT [28,29], are synthesized mainly in the liver and can reach the lungs by passive diffusion [30,31]. The point mutations in the gene that result in plasma deficiency of AAT or ACT are associated with COPD [32]. However, no polymorphisms have been reported for SLPI or elafin, and therefore, the question remains open as to whether a deficit in either SLPI or elafin contributes to the development of COPD in patients who otherwise have sufficient levels of AAT and ACT. On the other hand it has been suggested that increased plasma levels of other serine protease inhibitors, such as ACT and SLPI, might favourably improve the protease/anti-protease balance in subjects with severe AAT deficiency.

To further evaluated this hypothesis, we analysed plasma levels of AAT, ACT and SLPI and compared in both healthy and COPD adult patients with and without AAT deficiency. We analysed these inhibitors in two different groups of individuals, the first group consisting of 31 year old asymptomatic AAT deficiency individuals from the prospective follow-up study [20], and the second consisting of older asymptomatic and COPD subjects with severe AAT deficiency from the Malmö AAT Deficiency Register. Age and gender matched individuals with wild type MM AAT were used for comparison.

Reports published from the prospective follow-up study of ZZ and SZ individuals up to age 26 years, focusing on clinical health, lung and liver function tests and plasma markers of the protease/protease inhibitor balance, have shown that ZZ and SZ subjects had significantly higher plasma concentrations of α2-MG, ACT and antithrombin III [20] at age 8 and 18 compared with MM control subjects. Recent findings at age 26 for ZZ and SZ subjects with normal lung function and only marginal deviations in liver test results showed significantly higher plasma SLPI levels, but not α2-MG, compared to age matched healthy MM subjects [20]. In contrast, at age 31, we find in this study that ZZ and SZ individuals have no significant difference in plasma SLPI and ACT levels compared to MM controls. The higher plasma α2-MG, SLPI and ACT levels reported in AAT deficiency subjects at younger ages were previously attributed to an unidentified compensatory mechanism that protects lung tissue against proteolytic injury under conditions of AAT depletion. Our results show that this differential is not observed at age 31 and older, and raise the question of whether the marginal differences in protease inhibitor levels observed at younger ages between AAT deficient subjects and normal are of clinical importance.

The increased protease inhibitor plasma levels in young AAT deficiency subjects relative to normal subjects could be linked to age rather than to a ZZ or SZ phenotype. For instance, α2-MG levels in cord blood and in young children have been found to be very high and to fall fairly rapidly from age 15 to 20 years, and to continue to decline gradually until the age of 30 to 40 [33-35]. Similar measurements in another study found concentrations of α2 MG in normals to be high in youth, reach their minimum in middle age, and gradually increase with old age [36]. It has been shown that plasma α2 MG levels in ZZ AAT subjects dropped from 310% at age 8 years to 215% at 18 years and to normal 100% levels at adult age. This pattern tracked that observed in MM AAT control 8-year olds in whom the plasma concentrations of α2-MG and α2-antiplasmin were found to be increased up to 252% and 125% of adult levels (100% corresponding to 2.5 mg/ml and 70 μg/ml, respectively)[20] and to decline to normal at adult age. These data indicate an inherent elevation of α2-MG in the young that is not exclusive to the AAT deficient population.

It is also important to point out that, for example, when subjects were 26 years old, but not 18 and 31 years old, SLPI was found to be significantly higher in AAT-deficient subjects than in wild type controls. These inconsistent findings during the follow-up studies suggest that plasma may not be the relevant biological fluid to measure SLPI levels. Alternatively, because SLPI is produced locally in the airways and is regulated by various pro-inflammatory stimuli, its plasma levels when analysed in healthy (asymptomatic) subjects may not reflect the real situation. One can not exclude that under inflammatory conditions the compensatory increase in SLPI and/or other protease inhibitors may reduce the severity of AAT deficiency by favourably affecting the overall protease/protease-inhibitor balance in AAT-deficient individuals.

Our data show small increases in ACT and SLPI, as well as AAT, concentrations in normal MM subjects with COPD relative to well subjects. These differences exceed those for the same parameters for ZZ subjects with and without COPD and suggest that the COPD disease state could confound conclusions drawn from comparative studies seeking to attribute phenotypes (elevated protease inhibitor levels) to genotypes (ZZ, ZS AAT deficiency).

## Conclusion

We conclude that there is no clear evidence for compensatory up-regulation of protease inhibitors, such as ACT and SLPI, in healthy (asymptomatic) subjects with severe AAT deficiency. Thus, the changes in circulating levels of protease inhibitors and their likely impact on individual susceptibility to lung disease remains to be confirmed through further studies.

## Abbreviations

AAT, alpha-1-antitrypsin; PiZZ, homozygous AAT-deficiency variant; PiMM, wild type AAT variant; ACT, alpha-1-antichymotrypsin; COPD, chronic obstructive pulmonary disease; α2-MG, alpha2-macroglobulin; SLPI, Secretory leukocyte proteinase inhibitor; FEV_1_, forced expiratory volume in 1 second; FVC, forced vital capacity;

## Competing interests

The author(s) declare that they have no competing interests.

## Authors' contributions

CH carried out the analysis, participated in the interpretation of data and helped to draft the manuscript. EP and TS collected patient material. UW and AW helped with the interpretation of data and the study design. SJ designed the study, carried out the final statistical analysis and wrote the manuscript. All authors have read and approved the final manuscript.

## Pre-publication history

The pre-publication history for this paper can be accessed here:


